# Health and healthcare in North Korea: a retrospective study among defectors

**DOI:** 10.1186/s13031-020-00284-y

**Published:** 2020-06-29

**Authors:** Hayoung Lee, Courtland Robinson, Jaeshin Kim, Martin McKee, Jiho Cha

**Affiliations:** 1 Independent Consultant, Seoul, South Korea; 2 Center for Humanitarian Health, Johns Hopkins Bloomberg School of Public Health, Baltimore, USA; 3 Dankook Center for Dispute Resolution, Dankook University, Yongin, Republic of Korea; 4 London School of Hygiene and Tropical Medicine, London, UK; 5 Humanitarian and Conflict Response Institute, University of Manchester, Manchester, UK

**Keywords:** Health disparities, Access to healthcare, Political inequality, Health system, North Korea

## Abstract

**Background:**

To gain insights into the socio-economic and political determinants of ill health and access to healthcare in North Korea.

**Methods:**

A retrospective survey using respondent-driven sampling conducted in 2014–15 among 383 North Korean refugees newly resettling in South Korea, asking about experiences of illness and utilization of healthcare while in North Korea, analyzed according to measures of political, economic and human rights indicators.

**Results:**

Although the Public Health Act claims that North Korea provides the comprehensive free care system, respondents reported high levels of unmet need and, among those obtaining care, widespread informal expenditure. Of the respondents, 55.1% (95%CI, 47.7–63.7%) had received healthcare for the most recent illness episode. High informal costs (53.8%, 95%CI, 45.1–60.8%) and a lack of medicines (39.5%, 95%CI, 33.3–47.1%) were reported as major healthcare barriers resulting in extensive self-medication with narcotic analgesics (53.7%, 95%CI, 45.7–61.2%). In multivariate logistic regressions, party membership was associated with better access to healthcare (Adjusted OR (AOR) = 2.34, 95%CI, 1.31–4.18), but household income (AOR = 0.40, 95%CI 0.21–0.78) and informal market activity (AOR = 0.29, 95%CIs 0.15–0.50) with reduced access. Respondents who could not enjoy political and economic rights were substantially more likely to report illness and extremely reduced access to care, even with life-threatening conditions.

**Conclusions:**

There are large disparities in health and access to healthcare in North Korea, associated with political and economic inequalities. The scope to use these findings to bring about change is limited but they can inform international agencies and humanitarian organizations working in this unique setting.

## Background

The health system in the Democratic People’s Republic of Korea (DPRK, or North Korea) shares many features with those in place in other parts of the pre-1989 Communist bloc. Created as a state entity, it is funded by government revenues and delivered within an extensive network of well-staffed health facilities [1–3]. It has, in theory, provided universal coverage but the reality is now somewhat different. Although the country initially outperformed its Southern neighbor economically, by the 1970s its economy was struggling and, in 1979, it defaulted on many of its international debts. A further blow came in 1991, when the collapse of the USSR cut of economic support on which it depended [4]. Soon after, shortages of fuel and fertilizer, combined with widespread flooding, contributed to large scale famines [5]. The economy, a large share of which is now devoted to the military, has weakened further under pressure from international sanctions. The result is that the public distribution system (PDS), which allocated food along with goods such as clothes and household appliances to the population, has become dysfunctional, [6] and the centralized health system has been weakened from a chronic shortage of funds and a lack of essential medicines and medical supplies [7, 8]. An analysis of data from two censuses, in 1993 and 2008, suggests that life expectancy has fallen substantially, with the greatest contribution from rising deaths in childhood and late middle age [9]. Meanwhile, a rapidly expanding informal market mechanism has resulted in parallel economic systems for commercial distribution of essential items and the informal generation of household income mostly through an informal system of local markets, termed *Jangmadang* (literally translated as “market grounds”) filling the gaps created by the deteriorating Public Distribution system [10, 11]. In addition, a nascent informal market in healthcare has expanded [7] although the government still presents the socialist health system as the only one [1, 3].

These changes are almost certain to have impacted adversely on the experiences of North Koreans in need of healthcare. Yet, while the worsening economic situation and, especially, the intensification of sanctions has attracted media attention, especially in relation to food supply, the consequences for healthcare are much less clear. A rare exception was a widely publicized case of extensive parasitic worm infection in a defector, reported as indicative of health system failings, although commentators noted that generalization from a single case is unwise [12].

Perhaps unsurprisingly, there has been relatively little published by the UN and other international organizations working in North Korea [1, 3, 13]. There are obvious problems in obtaining direct access to the North Korean population and the few sources of data [14] are extremely sparse. Given the nature of the regime, it is difficult to assess their validity and representativeness.

This challenge resembles that facing western researchers in the post-war period who were seeking information on the Soviet health system. This stimulated a major program of interviews with defectors [15]. Although the methods employed were, by current standards, problematic, this did yield important insights into the prevailing situation that were unobtainable from other sources [16]. Drawing on this tradition, we conducted a retrospective survey among North Korean refugees and migrants covering the five-year recall period prior to their displacement, in collaboration with the US Centers for Disease Control and Prevention (CDC) and the Korean Institute for National Unification (KINU).

## Methods

In September 2014 and January 2015, a Respondent-Driven Sampling (RDS) design was used to contact North Korean adults newly resettling in urban communities in South Korea [17]. These individuals are not easily accessible due to their concerns about collective punishment of their families left behind in North Korea. RDS is designed to reduce some of the biases associated with traditional chain referral sampling and has been successfully used in various hard to reach populations, including urban refugees [18–21]. Following key informant interviews, we selected ten North Korean refugees as recruiter seeds and provided them with coupons to recruit other eligible participants from their social network. We limited each recruiter to three coupons to minimize any selection bias that might arise from the use of the initial seeds. Every new participant that completed the study survey was given three coupons for further recruitment (wave two). The recruitment waves were repeated until reaching equilibrium (wave nine), defined as when the composition of key variables, such as the sex ratio, ceases to change in the sample [22]. We traced referral patterns (who recruited whom) using coupon numbers and the size of social networks of participants in refugee communities. Potential bias from different network size and referral patterns were weighted with the RDS data analysis software (RDSAT, version7.1).

Following exploratory qualitative interviews with 34 North Koreans, we developed a structured questionnaire with both open and close-ended items that took 60–90 min to complete. For self-reported morbidity and healthcare access, we first asked about any illnesses of respondents occurring during the year prior to leaving North Korea, and any life-threatening illness (respondent) or death (family) where it was not possible to access healthcare. We also asked whether they received appropriate and/or free medical services in their most recent illness episode. Exposure to life-threatening cold was also asked. Lastly, we collected detailed information on the most recent episode of access to healthcare, including the nature of episodes of illness, direct costs (what and how to pay), medicines (what and where obtained), types of barriers, self-treatment, and drug and substance abuse.

Using a Human Rights Violation Inventory in North Korea (HRVI-NK), developed for this study, we collected data on respondents’ exposure to political and civil rights violations (19 items Cronbach α = 0.83) including torture and inhuman treatment (2 items, α = 0.80); discrimination (3 items, α = 0.47); freedom of movement and residence (4 items, α = 0.59); freedom of thought, expression and religion (6 items, α = 0.71); and arbitrary arrest, enforced disappearance and detention (4 items, α = 0.56). Human rights violations related to forced labor (3 items, α = 0.63) and rights to livelihood (2 items, α = 0.46) were used as additional indicators of social and economic rights violations.

Data on political status and socioeconomic position were obtained, along with basic demographic information. Political status in North Korea was measured by *Songbun* system, and a membership of the Worker’s Party Korea (WPK) [11]. The *Songbun* system classifies people according to the characteristics of their ancestors and the behavior of their relatives. People are defined as “core”, “wavering”, or “hostile”, with the first including those whose ancestors fought against the Japanese and the last including those where they were merchants or Christian ministers, for example. Economic status was assessed by an asset index, derived using principal components analysis (PCA) with household ownership of 14 consumer items identified during formative interviews. Spearman’s rank correlation coefficients for political and economic variables were calculated, finding no significant correlation among them. In addition, we asked whether respondents and/or household members were engaged in black market trading. Lastly, we collected patterns of displacement.

We fully recognize the risk of recall bias when asking about detailed and complex information. While the most recent episode of illness and healthcare utilization were asked about, we limit the recall period to the five years prior to displacement. If the respondent has no experience of illness or healthcare within the recall time, or cannot remember it, those questions were skipped. A five year recall period is used in several large-scale population-based household surveys in low and middle income countries [23]. For human rights violations, however, we exceptionally consider 10 years recall time due to the intensity of the potentially traumatic memory [17].

Key estimates of interest and confidence intervals (95% CI) were adjusted for Respondent-Driven Sampling using RDSAT version 7.1. Standard exploratory data analysis procedures were to explore descriptive components that involve detailed patterns of healthcare utilization and key health indicators. Homophily (range: − 1[heterogeneous], 1[homogenious]), the tendency of people to recruit people similar to themselves, was assessed with key variables of interest. A multivariate logistic regression model was undertaken to identify associations of healthcare access, morbidity, and health status with political and economic status and human rights experiences, adjusted for key socio-demographic factors. The initial ten seeds were not included in the analysis.

### Ethical consideration

The study protocol was approved by the Institutional Review Board (IRB) of Dankook University in Korea and, as secondary data analysis, by the Johns Hopkins Bloomberg School of Public Health in Baltimore, Maryland. With RDS, all participation is voluntary, but we made every effort to maximize protection and confidentiality of North Korean participants. The study objectives, survey items, and potential risks of participation were explained prior to the interviews, and informed consent was obtained. We scheduled an interview at a location chosen by the participant. Each interview was administered by North Korean refugee surveyors who were trusted by the local refugee community and had proven experience conducting surveys. Participants received KRW 20,000 (US$18) in compensation for their time and transportation expenses.

## Results

Subtracting the ten initial seeds, a total of 383 North Korean refugees and migrants participated in the survey. Of the participants, 71.8% (95% CI, 67.0–76.2%) were female, and 28.2% (95% CI, 23.8–33.0%) were male. This is almost identical to the sex ratio of the entire North Korean population resettled in South Korea since 1999 (71.8% women and 28.2% men) [24]. Around one third (31.0%) had been members of the WPK and 67.3% had a family member who was a member. Two thirds (64.0%) had lived in extreme poverty with a household income under US$1 per day. However, most participants had a middle school or higher level of education. While only 2.1% were educated to primary school or lower, 16.4% had a university education. A total of 74.8% came from urban areas in North Korea.

Even though the Public Health Act in North Korea claims that it provides complete and comprehensive free care, out of pocket expenditures for health services were widespread, especially for medical consultations (65.4%), medicines and medical supplies (82.0%), and other items such as meals or heating when in health facilities (75.9%) (here and below we report figures adjusted for the RDS method). (Table 1) Most respondents reported that they purchased medicines and medical supplies from pharmacies (60.5%) or street stalls (42.5%) in the informal market. Only 10.0% received them from hospitals or clinics in the formal sector. Respondents could make informal payments, in cash, obtained from black market employment (47.3%) or the sale of household items (39.8%), while only 7.1% reported the public distribution system as their source of income for medical expenditure. Among those reporting not accessing health services when it was needed, 30.1% reported receiving neither a diagnosis nor treatment. Half (52.1%) reported self-medication without a diagnosis, and 18.2% received a diagnosis but did not receive any treatment. Pharmacy (55.4%) and street stalls (40.8%) in the informal market were major sources of self-treatment, followed by using traditional medicine (10.6%) or taking left over (7.7%) or other person’s medicines (3.0%). The primary reasons for not seeking care were medical costs (53.8%) and a lack of medications and medical supplies in the health facilities (39.5%).

**Table 1:** Health service utilization and barriers experienced by respondents in North Korea (Crude and adjusted for RDS methodology)

		Crude	Adjusted
		Freq (%)	% [95% CI]
**HEALTH SERVICE UTILIZATION**^a^			
Medical costs	Medical consultation	186 (62.6)	65.4 [58,74.1]
	Medicine and medical supplies	228 (79.4)	82.0 [75.5,88.1]
	Others (e.g., meals or heating)	162 (72.6)	75.9 [65.7,84.1]
	Bribe	152 (56.7)	57.8 [49.2,66.4]
Resource for payment	Black market employment	124 (46.8)	47.3 [40.4,55.3]
	Public distribution system	19 (7.2)	7.1 [3.5,11.5]
	Sale of household items	89 (33.6)	39.8 [30.9,47.3]
	Borrowed	38 (14.3)	17 [10.4,23.3]
	Support of relatives or neighbors	57 (21.5)	19.4 [13.1,26.3]
Location of the purchase of medicines & medical supplies	Informal market (pharmacy)	179 (59.1)	60.5 [53.2,66.9]
	Informal market (street stalls)	125 (41.3)	42.5 [35.8,49.9]
	Hospital or clinic	39 (12.9)	10.0 [6.8,15.1]
Type of medication	Formal medicine	236 (77.6)	79.5 [73.2,84.6]
	Narcotic analgesics	119 (39.1)	39.7 [32.5,49.3]
	Methamphetamines	7 (2.3)	1.8 [0.4,4.1]
**HEALTH SERVICE BARRIERS**^b^			
Types of healthcare services not received	No diagnosis and treatment	86 (28.7)	30.1 [23.7,36]
	Self-medication without diagnosis	148 (49.3)	52.1 [44.9,58.2]
	Diagnosis without treatment	60 (20)	18.2 [13.9,24]
Reason for not receiving care	Medical costs	145 (49.2)	53.8 [45.1,60.8]
	Lack of medicine and medical supplies	118 (40)	39.5 [33.3,47.1]
	Closed clinic and hospital	28 (9.5)	10.9 [5.7,16.1]
	No trust in health professional	28 (9.5)	10 [6.5,15.5]
	Physical distance to clinic	25 (8.5)	7.7 [4.3,12.9]
Source of self-treatment	No treatment	34 (11.2)	10.1 [6.3,13.4]
	Informal market (pharmacy)	148 (49)	55.4 [48.4,62.9]
	Informal market (street stalls)	122 (40.4)	40.8 [34.1,49.3]
	Remaining medications	29 (9.6)	7.7 [4.2,11.4]
	Traditional medicine	43 (14.2)	10.6 [6.7,15]
Type of self-medication	Formal medicine	184 (63)	63.5 [57.1,71]
	Narcotic analgesics	163 (55.8)	53.7 [45.7,61.2]
	Methamphetamines	7 (2.4)	2.7 [0.3,6.2]

^a^Most recent case of access to healthcare within 5 years prior to displacement

^b^Most recent case of barrier to healthcare within 5 years prior to displacement

The prevalence of drug and substance misuse was high, with 53.7% reporting self-medication narcotic analgesics and 2.7% with methamphetamines. Medicines such as NSAIDs, antibiotics, anti-TB drugs were widely used without preceding medical consultations (63.5%).

Only half of respondents (55.1%) had received healthcare for their most recent illness episode (Table 2). Of them, 31.9% reported that they needed to bribe health professionals or other individuals to receive services, although 37.8% were able to obtain formal health services free of charge the last time it was needed. There were political and economic disparities in self-reported morbidity and healthcare access, as described below.

**Table 2:** Self-reported morbidity and access to health in North Korea

	Crude	Adjusted
	Freq (%)	% [95% CI]
**Morbidity**		
Illness within 1 year before displacement	227 (62.9)	62.4 [55.9,68.7]
Life threatening illness without healthcare^a^	158 (42.4)	40.1 [34.6,45.9]
Life threatening due to severe cold^a^	120 (32.2)	33.0 [26 to 39.8]
**Access to healthcare**		
Health service, last illness episode	206 (57.7)	55.1 [47.7,63.7]
Health service, free of charge (formal sector)	149 (43.7)	37.8 [30.6,45.6]
Health service, bribed (formal sector)	116 (33.8)	31.9 [25.5,38.9]

^a^These life-threatening events were collected with 10 years recall period

### Multivariate logistic regression

The multivariate logistic regression employed two models, the first adjusting for political and economic status variables along with a range of sociodemographic variables (Table 3). The second, which in addition examined political and economic rights, was fully adjusted for all of the variables falling in these categories as well as those relating to political, economic and sociodemographic status (Table 4).

**Table 3:** Multivariate logistic regression model 1 (weighted): health and healthcare associated with political and economic status

Model 1: Multivariate logistic regression^a^	Household Health		Healthcare access		
	Morbidity	Mortality^b^	Healthcare Access	Life-threatening illness without care	Life-threatening exposure to cold
**Political Status**	AOR [95%CI]	AOR [95%CI]	AOR [95%CI]	AOR [95%CI]	AOR [95%CI]
Workers’ Party of Korea member	0.91 [0.52,1.59]	0.71 [0.39,1.29]	2.34** [1.31,4.18]	0.81 [0.47,1.39]	1.10 [0.64,1.88]
**Economic Status**					
Household wealth (Top 40% vs bottom 20%)	1.18 [0.52,2.67]	3.41* [1.12,10.36]	0.36* [0.15,0.86]	2.44* [1.10,5.40]	2.15 [0.93,4.95]
Household income (below $1/Day)	0.83 [0.43,1.62]	1.05 [0.49,2.24]	0.40** [0.21,0.78]	0.53 [0.28,1.00]	1.62 [0.83,3.15]
Market business	2.50*** [1.45,4.31]	1.82 [0.95,3.53]	0.29*** [0.15,0.50]	1.29 [0.74,2.23]	1.90* [1.08,3.31]

^a^Multivariate logistic regression model 1: Health outcomes (dependent variables) associated with a membership of Workers Party Korea; Household Wealth, Poverty; Informal Market Business, adjusted for demographic variables (Gender, Age, Edu, Region of Residence)

^b^Respondent reporting death of family member due to life threatening illness without care

*p < .05; **p < .01; ***p < .001

**Table 4:** Multivariate logistic regression model 2 (weighted): health and healthcare associated with political and economic rights violations

Model 2: Multivariate logistic regression^a^	Household Health		Access to Healthcare		
	Morbidity	Mortality^b^	Healthcare Access	Life-threatening illness without care	Life-threatening exposure to cold
**Political and Civil Rights**	AOR [95%CI]	AOR [95%CI]	AOR [95%CI]	AOR [95%CI]	AOR [95%CI]
Torture & inhumane treatment	1.18 [0.67,2.06]	1.90* [1.06,3.43]	1.04 [0.60,1.83]	3.13*** [1.82,5.37]	2.25** [1.33,3.80]
Discrimination	1.82* [1.09,3.06]	4.45*** [2.37,8.38]	0.64 [0.38,1.08]	2.23** [1.34,3.71]	2.26** [1.36,3.76]
Freedom of movement & residence	3.01*** [1.69,5.38]	5.00*** [1.98,12.65]	0.39** [0.21,0.74]	3.23*** [1.64,6.36]	2.90** [1.53,5.51]
Freedom of thought, expression, & religion	1.80* [1.04,3.11]	5.18*** [2.29,11.72]	1.01 [0.58,1.76]	4.28*** [2.29,8.00]	3.51*** [1.93,6.36]
Arbitrary arrest, disappearance, & detention	2.69*** [1.54,4.70]	5.69*** [2.65,12.24]	0.33*** [0.18,0.59]	8.44*** [4.29,16.62]	3.29*** [1.85,5.84]
**Social and Economic rights**					
Forced labor	1.86* [1.05,3.29]	9.93*** [3.19,30.92]	0.50* [0.27,0.92]	7.95*** [3.50,18.06]	2.41** [1.31,4.43]
Rights to Livelihood	4.02*** [2.32,6.96]	4.03*** [2.11,7.69]	0.48** [0.28,0.82]	8.36*** [4.44,15.74]	6.77*** [3.78,12.12]

^a^Multivariate logistic regression model 2: Health outcomes associated with political and economic rights violations (dependent variables) adjusted for a membership of Workers Party Korea; Household Wealth; Poverty; Informal Market Business, and demographic variables (Gender, Age, Edu, Region of Residence)

^b^Respondent reporting death of family member due to life threatening illness without care

*p < .05; **p < .01; ***p < .001

In the first model the probability of reporting illness in the year prior to displacement was more than doubled among those who had been employed in the black market. In the second model, this probability was increased substantially in those experiencing each of the violations of political and civil rights enquired about, with the exception of torture, and in those who had suffered violation of their economic rights. Among these associations, the most important were restrictions on freedom of movement and residence and the right to livelihood. Also, the probability of reporting deaths of family member is significantly higher in the lowest household wealth group, and those who experiencing any of political and economic rights violations.

Turning to access to care during the last illness, in the first model (Table 3), members of the Workers’ Party of Korea were almost three times as likely to have access to care than those who were not members. Those who were poor or who worked in the black market were about one third as likely to have access to care as those who were not in these categories. In the second model (Table 4), access was reduced in those who have experienced restrictions on movement and residence, arbitrary arrest, restrictions on the right to livelihood, and forced labor.

The numbers of respondents experiencing life-threatening illness were, inevitably, somewhat smaller. However, those experiencing violation of a range of political and economic rights were very much more likely to have lacked care when needed, in some cases up to eight times more than those whose rights had not been violated (Table 4). Among this sample, furthermore, those who had experienced violations of their political and economic rights were substantially more likely to have been exposed to the cold. However, with the exception of involvement in the black market, there was no correlation with the political and economic status variables.

In summary, these results confirm the privileged status of members of the party in their ability to access healthcare. Those working in the black market are at increased risk of illness and, when they become ill, seem less able to obtain healthcare and shelter. Those whose political rights are being violated are not only more likely to have experienced illness and death of family members but much less likely to have accessed healthcare, especially if they have experienced life-threatening illness, or to have been protected from the cold. The situation is especially bad for those who have experienced arrest or forced labour.

## Discussion

The almost unique circumstances in North Korea make the usual approaches to assessing population health impossible. As in the post-war USSR, the only feasible approach is to undertake indirect sampling of North Korean refugees and migrants, with Respondent-Driven Sampling methods (RDS) used to access this hard to reach population. There are obviously many limitations, discussed below, but these North Koreans were willing to provide extensive, detailed, and consistent accounts of their experiences of health service utilization prior to displacement, encouraged by a combination of assured anonymity and relative safety outside North Korea.

Inevitably, caution is needed in extrapolating these results. First, survival bias is substantial in a retrospective study of the migrant population, which will almost inevitably result in under-representation of those who are unable to move easily, such as political prisoners, drug users or those with major illnesses. Meanwhile, refugees who are prepared to risk the dangers of escaping may be more likely to be socially marginalized than others in North Korea, and their experience could be over-represented in the sample. The refugee/migrants, thus, cannot fully represent the entire population in North Korea. Second, RDS does not generate a fully random sample representative of the refugee population, and limitations inherent in RDS methods all apply to this study [25–27]. Third, as a cross-sectional retrospective survey, it is impossible to determine a sequence of events, thus limiting any attempt to assess causality.

In the absence of alternative sources, these data add to our extremely limited understanding of the changing health system in North Korea. Previously, most attention had been directed to regional differences in access to healthcare, with politically important areas, such as the capital, *Pyongyang*, privileged over remote areas, especially the Northern Provinces [28, 29]. International humanitarian and development actors had tried to reach these remote areas but faced constraints imposed by the North Korean authorities. Yet, as in many parts of Asia and Africa, [30] the rapid expansion of informal health markets is believed to have improved availability of health services and medicines in these once marginalized areas. Now, the major concerns relate to growing economic inequalities, created by the increase in market mechanisms that circumvent the PDS. Private payments for healthcare outside the state system are now common. As a consequence, informal out of pocket payments have emerged as important barriers to obtaining health services. Only half of our respondents reported they could access to health services the last time it was needed. Illness and lack of healthcare were clearly more common in those living in poorer households and those depending on informal market function outside the state system.

It might be expected that political factors would influence access to services, something that these findings confirm, with clear differences between those politically privileged and disadvantaged. First, while the state’s monopoly on essential items such as pharmaceutical products and medical equipment has been weakened with the malfunctioning of the PDS, to the extent that it does function at all, it still privileges individuals on the basis of their political class, using the *Songbun* system, described earlier [29, 31, 32]. This system has become less important in recent years but it was notable that those with a family member in the WPK had still better access to the formal health system. In many transitioning societies, political capital attached to individuals or those in certain social groups offers privileged access to resources, influence, and peer support, while reducing exposure to collective and interpersonal violence [33]. These all act as drivers of inequities in health. We found clear evidence of political inequalities in health and healthcare. Those who could not enjoy political and civil rights were more likely to report illness and extremely reduced access to care, even if they had life-threatening conditions. Political inequality, with the consequent disproportionate distribution of power, might be unavoidable in real world. Its adverse consequences for health and healthcare, however, should be addressed and mitigated.

## Conclusions

While recognizing the many limitations that are inevitable when doing research in these very difficult circumstances, as far as we are aware our findings pointing to the scale and nature of disparities are unique, going beyond anything that has been reported from studies conducted by North Korean and international agencies [1, 2, 34]. Our findings paint a picture in which many North Koreans now struggle to obtain healthcare, in a situation characterized by a weakened state system and a growing unregulated informal market. Some are able to navigate the system, but many are not, disadvantaged by their exclusion from the political and economic mainstream.

Health research should inform policy but it is self-evident that these findings are unlikely to achieve any substantial impact with the current North Korean government. However, even now, there are several international humanitarian organizations working in the country, as well as international agencies such as the WHO. We hope that they, at least, will find these results useful in their work on the ground and their dialogues with the North Korean authorities.

## Data Availability

The datasets generated and/or analyzed during the current study are not publicly available due to the potential security risk of raw data* but are available from the corresponding author on reasonable request. *Our interviewees, North Korean refugees, have concerns of potential political stigma of their left-behind families in North Korea, when they participated in our epidemiological study of human rights violations. Human rights are extremely sensitive issues for North Korean government. Even if our raw data does not include any personal identification information, research participants have considerable fears about releasing their individual data set in public without any restriction. For them, it may mean that North Korean government can also access the raw data freely. Given the ethical and security concerns on this fear, we think that minimum restrictions need to be applied in public availability of this rare and important data.
